# Comparison of High, Intermediate, and Low Frequency Shock Wave Lithotripsy for Urinary Tract Stone Disease: Systematic Review and Network Meta-Analysis

**DOI:** 10.1371/journal.pone.0158661

**Published:** 2016-07-07

**Authors:** Dong Hyuk Kang, Kang Su Cho, Won Sik Ham, Hyungmin Lee, Jong Kyou Kwon, Young Deuk Choi, Joo Yong Lee

**Affiliations:** 1 Department of Urology, Severance Hospital, Urological Science Institute, Yonsei University College of Medicine, Seoul, Korea; 2 Department of Urology, Gangnam Severance Hospital, Urological Science Institute, Yonsei University College of Medicine, Seoul, Korea; 3 Division of Epidemic Intelligence Service, Korea Centers for Disease Control and Prevention, Osong, Korea; 4 Department of Urology, Severance Check-Up, Yonsei University Health System, Seoul, Korea; National Taiwan University, TAIWAN

## Abstract

**Objectives:**

To perform a systematic review and network meta-analysis of randomized controlled trials (RCTs) to determine the optimal shock wave lithotripsy (SWL) frequency range for treating urinary stones, i.e., high-frequency (100–120 waves/minute), intermediate-frequency (80–90 waves/minute), and low-frequency (60–70 waves/minute) lithotripsy.

**Materials and Methods:**

Relevant RCTs were identified from electronic databases for meta-analysis of SWL success and complication rates. Using pairwise and network meta-analyses, comparisons were made by qualitative and quantitative syntheses. Outcome variables are provided as odds ratios (ORs) with 95% confidence intervals (CIs).

**Results:**

Thirteen articles were included in the qualitative and quantitative synthesis using pairwise and network meta-analyses. On pairwise meta-analyses, comparable inter-study heterogeneity was observed for the success rate. On network meta-analyses, the success rates of low- (OR 2.2; 95% CI 1.5–2.6) and intermediate-frequency SWL (OR 2.5; 95% CI 1.3–4.6) were higher than high-frequency SWL. Forest plots from the network meta-analysis showed no significant differences in the success rate between low-frequency SWL versus intermediate-frequency SWL (OR 0.87; 95% CI 0.51–1.7). There were no differences in complication rate across different SWL frequency ranges. By rank-probability testing, intermediate-frequency SWL was ranked highest for success rate, followed by low-frequency and high-frequency SWL. Low-frequency SWL was also ranked highest for low complication rate, with high- and intermediate-frequency SWL ranked lower.

**Conclusions:**

Intermediate- and low-frequency SWL have better treatment outcomes than high-frequency SWL when considering both efficacy and complication.

## Introduction

Since the introduction of shock wave lithotripsy (SWL) in the early 1980s, SWL has become a safe and accepted treatment modality for most intra-renal stones and many ureteral stones [1]. Despite the popular use of SWL, controversy remains regarding its success rate and the optimal shock wave (SW) frequency to achieve stone-free status. *In vitro* and animal studies have demonstrated that stone disintegration is influenced by the rate of SW administration, and slowing the rate to less than 120 SW/minute may improve stone fragmentation [2,3]. However, few clinical studies have evaluated the effect of varying SW frequency on stone fragmentation efficiency in humans [4,5].

The newly introduced network meta-analysis is a meta-analysis approach in which multiple treatments are compared using direct comparisons of interventions within randomized controlled trials (RCTs), and indirect comparisons are performed across trials based on a common comparator [6–9]. We performed a systematic review and network meta-analysis of RCTs to decide the optimal SW frequency range for disintegrating urinary stones by SWL. Frequency ranges were defined as high-frequency (100–120 SWs/minute), intermediate-frequency (80–90 SWs/minute), and low-frequency (60–70 SWs/minute).

## Materials and Methods

### Inclusion Criteria

Published RCTs that were in accordance with the following criteria were included: (i) Study design assessed different SW frequency ranges (100–120, 80–90, and 60–70 SWs/minute) to treat urinary tract stone disease. (ii) Baseline characteristics of patients from two or more groups were matched, including the total number of subjects and the values of each index. (iii) Outcomes of SWL were analyzed by stone-free or success rate according to each group. (iv) Standard indications for SWL to treat urinary tract stone disease were accepted. (v) Endpoint outcome parameters also included complication rate. (vi) The full text of the study was available in English. This report was prepared in compliance with the Preferred Reporting Items for Systematic Reviews and Meta-Analyses (PRISMA) statement (accessible at http://www.prisma-statement.org/) [10]. A protocol for this study was shown in S1 Table.

### Search Strategy

A literature search of all publications before 31 May 2015 was performed in EMBASE and PubMed. Additionally, a cross-reference search of eligible articles was performed to identify studies that were not found during the computerized search. The proceedings of appropriate meetings were also searched. Combinations of the following MeSH terms and keywords were used: extracorporeal shock wave lithotripsy, shock wave lithotripsy, frequency, renal stone, ureter stone, urolithiasis, success rate, stone-free, and randomized controlled trial (S2 Table).

### Data Extraction

A researcher (DHK) screened all titles and abstracts identified by the search strategy. Two other researchers (KSC and WSH) independently evaluated the full text of each paper to determine whether a paper met the inclusion criteria. Disagreements were resolved by discussion until a consensus was reached or by arbitration mediated by another researcher (JYL).

### Quality Assessment for Studies

After the final group of papers was agreed upon, two researchers (DHK and KSC) independently evaluated the quality of each article. The Cochrane Collaboration risk-of-bias as a quality assessment tool for RCTs was used. This assessment includes assigning a judgment of “yes”, “no”, or “unclear” for each domain to designate a low, high, or unclear risk of bias, respectively. If ≤1 domain was deemed “unclear” or “no”, then the study was classified as having a low risk of bias; if 2–3 domains, then moderate risk of bias; and if ≥4 domain, then a high risk of bias [11]. Quality assessment was performed with Review Manager 5 (RevMan 5.2.11 software, Cochrane Collaboration, Oxford, UK).

### Heterogeneity Tests & Inconsistency Assessment

Heterogeneity of included studies was examined using the Q statistic and Higgins’ I^2^ statistic [12]. Higgins’ I^2^ measures the percentage of total variation due to heterogeneity rather than chance across studies. Higgins’ I^2^ was calculated as follows:

$$I^{2}=\frac{Q-df}{Q}\times 100\%,$$

in which “Q” is Cochran's heterogeneity statistic, and “df” is the degrees of freedom.

An I^2^ ≥ 50% is considered to represent substantial heterogeneity [13]. For the Q statistic, heterogeneity was deemed to be significant for p<0.10 [14]. If there was evidence of heterogeneity, the data were analyzed using a random-effects model. Studies in which positive results had been confirmed were assessed with a pooled specificity using 95% CIs. In addition, L’Abbe plot and Galbraith’s radial plot were created to evaluate heterogeneity [15,16]. To assess inconsistency in the network, Cochran’s Q statistic and a net-heat plot were used and developed by Krahn et al. [17]. The net-heat plot is a graph that helps to identify pairwise comparisons that might be potential sources of important inconsistency in the network. A node-splitting analysis of inconsistency was applied in the forest plots of a network meta-analysis [18].

### Statistical Analysis

Outcome variables measured at specific time points were compared in terms of odds ratios (OR) or mean differences with 95% CIs using a network meta-analysis. Analyses were based on non-informative priors for effect sizes and precision. Convergence and lack of auto-correlation were confirmed after four chains and a 50,000-simulation burn-in phase; finally, direct probability statements were derived from an additional 100,000-simulation phase. The probability that each group had the lowest rate of clinical events was assessed by Bayesian Markov Chain Monte Carlo modeling. Sensitivity analyses were performed by repeating the main computations with a fixed-effect method. Model fit was appraised by computing and comparing estimates for deviance and deviance information criterion. All statistical analyses were performed with Review Manager 5 and R (R version 3.2.5, R Foundation for Statistical Computing, Vienna, Austria; http://www.r-project.org), the latter with associated netmeta, and gemtc packages for pairwise and network meta-analyses.

## Results

### Eligible Studies

**Fig 1:**
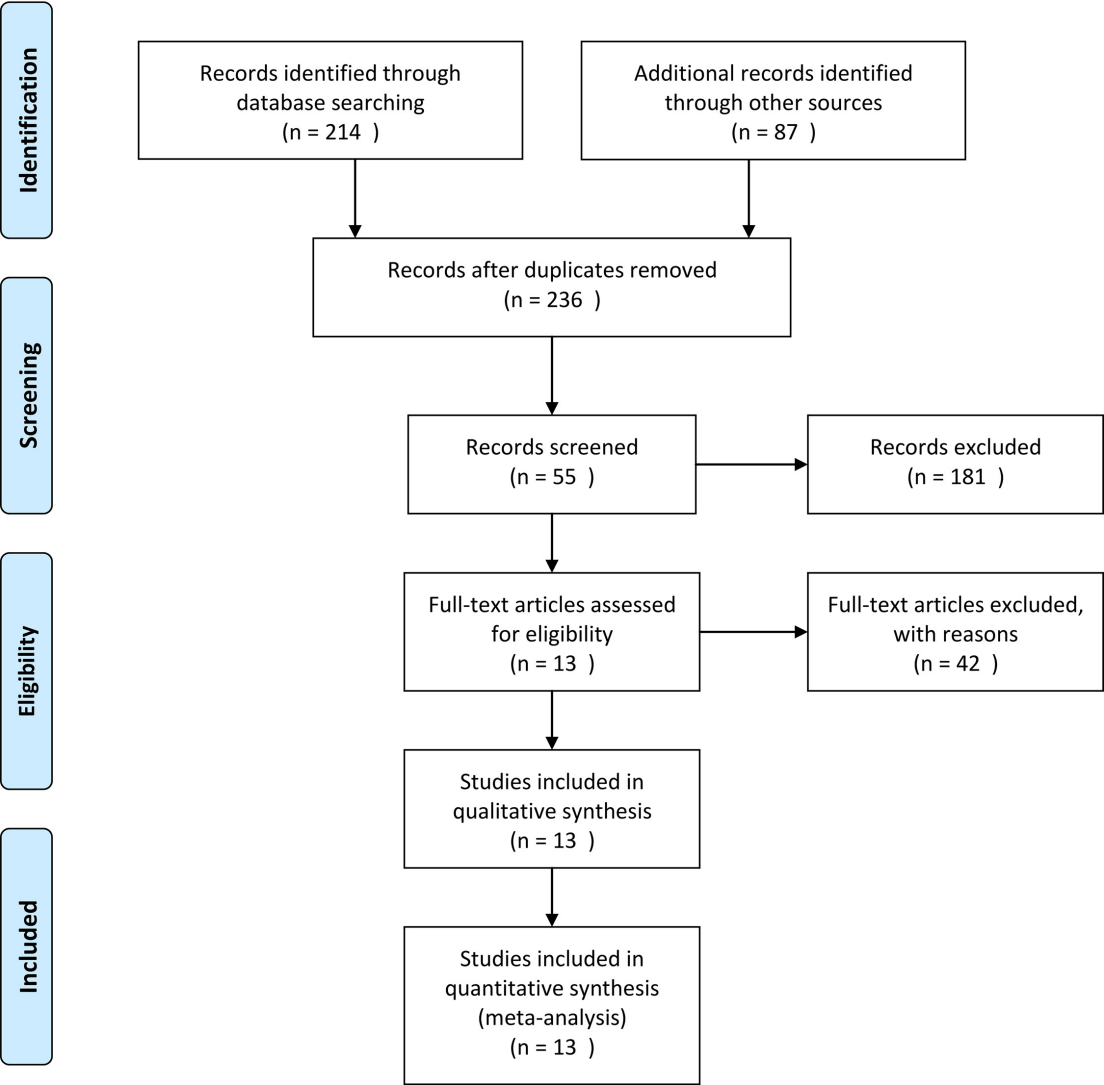
Flow diagram of evidence acquisition. Thirteen studies were ultimately included in the qualitative and quantitative synthesis that used pairwise and network meta-analyses.

The database search retrieved 55 articles covering 236 studies for potential inclusion in the meta-analysis. Forty-two articles were excluded according to the inclusion/exclusion criteria; 28 articles were retrospective models, 11 articles were reviews, and 5 articles were reported as case series. The remaining 13 articles were included in the qualitative and quantitative synthesis using pairwise and network meta-analyses (Fig 1).

**Fig 2:**
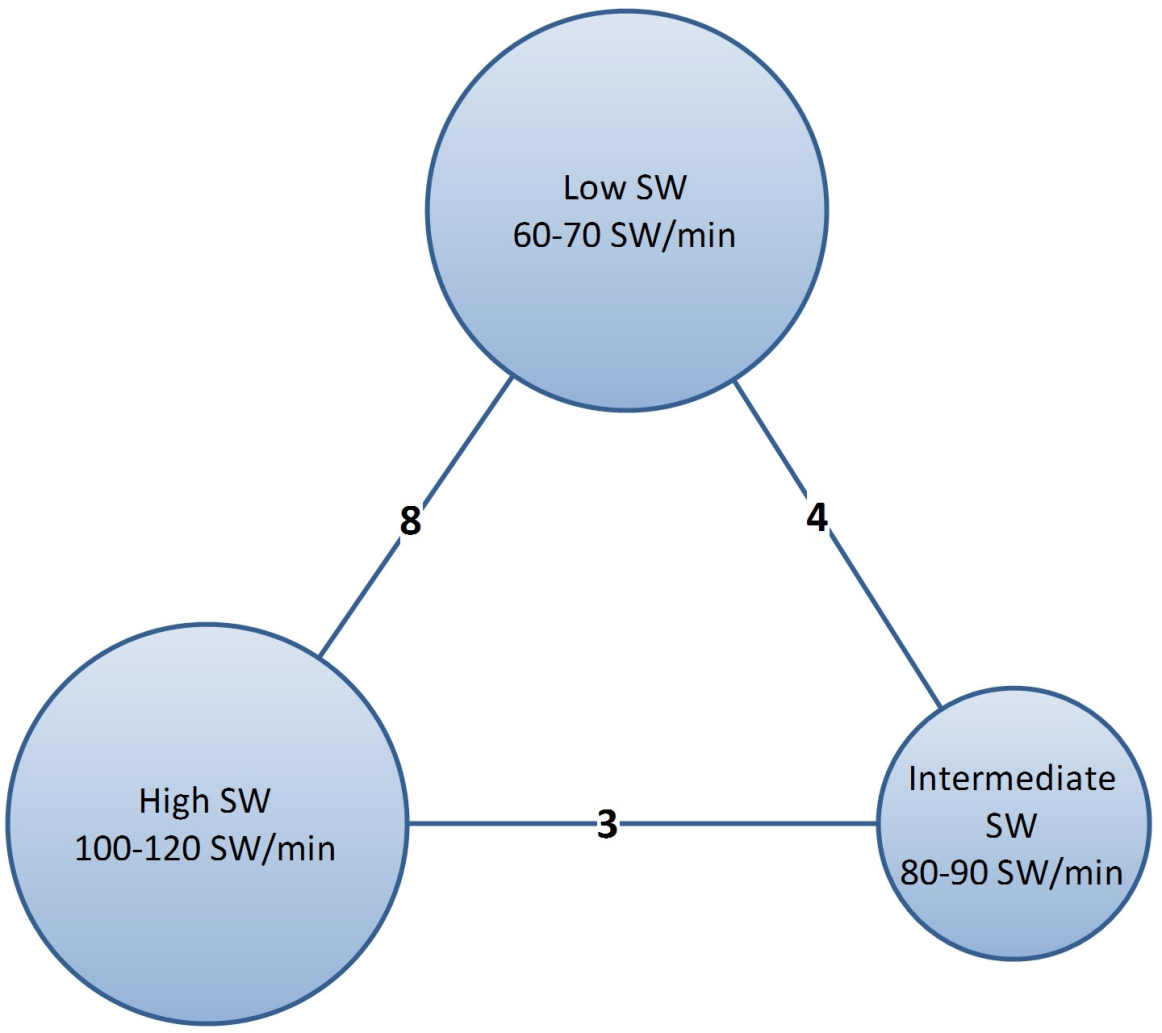
Network plots for included studies. Eight studies compared low-shock wave (SW) versus high-SW frequency ranges. Four trials reported outcomes comparing low-SW versus intermediate-SW frequencies. Three studies compared outcomes between high-SW and intermediate-SW frequencies.

Data corresponding to confounding factors derived from each study are summarized in Table 1. Eight studies compared low-frequency SWL versus high-frequency SWL [5,19–25]. Four trials reported outcomes between low- versus intermediate-frequency SWL [20,26–28]. Three studies compared outcomes between high- and intermediate-frequency SWL [20,29,30] (Fig 2). We summarized the success and complications of enrolled studies in Table 2.

**Table 1:** Enrolled studies for this meta-analysis.

Study	Year	Location	Size	Lithotripter Models	Type of lithotripter	Frequency(SW/min)	Frequency Category^a^	No. of Patients	No. of Sessions	Follow-up Duration	Bias Risk^b^	Modified Bias Risk^c^
Pace et al [19]	2005	Renal	N/A	LithoTron (HealthTronics, Marietta, Georgia)	electrohydraulic	60	Low	111	1	3 months	High	Moderate
						120	High	109	1			
Yilmaz et al [20]	2005	Renal	< 20 mm	Stone Litho3pter (PCK, Ankara, Turkey); electrohydraulic lithotripter	electrohydraulic	60	Low	57	1	10 days	High	Moderate
						90	Intermediate	57	1			
						120	High	56	1			
Madbouly et al [5]	2005	Renal or ureteral	≤ 30 mm	electromagnetic Siemens Lithostar Multi-line lithotripter	electromagnetic	60	Low	76	1 to 3	3 months	High	Moderate
						120	High	80	1 to 3			
Davenport et al [21]	2006	Renal	N/A	Dornier Lithotripter S	electromagnetic	60	Low	49	1	3 months	High	Moderate
						120	High	51	1			
Li et al [29]	2007	Renal or ureteral	≤ 30 mm	electromagnetic Siemens Lithostar Multi-line lithotripter (Siemens, Munich, Germany)	electromagnetic	90	Intermediate	57	1	4 weeks	High	Moderate
						120	High	59	1			
Honey et al [22]	2009	Upper ureteral	≥ 5 mm	Philips LithoTronTM spark gap lithotripter	electrohydraulic	60	Low	77	1	3 months	High	Moderate
						120	High	86	1			
Koo et al [23]	2009	Renal	≤ 20 mm	Model S, Dornier MedTech, Wessling, Germany	electromagnetic	70	Low	51	1.3	≥ 6 months	Moderate	Low
						100	High	51	1.7			
Mazzucchi et al [26]	2010	Renal or ureteral	> 6 mm or symptomatic ≤ 6 mm	Dornier Compact Delta lithotripter	electromagnetic	60	Low	143	N/A	3 months	Moderate	Low
						90	Intermediate	157	N/A			
Chang et al [25]	2012	Renal	5 to 20 mm	Sonolith Praktis lithotripter (EDAP)	electroconductive	60	Low	81	N/A	3 months	High	Moderate
						120	High	80	N/A			
Ng et al [24]	2012	Renal	5 to 20 mm	Sonolith Vision (EDAP)	electroconductive	60	Low	103	N/A	12 weeks	High	Moderate
						120	High	103	N/A			
Anglada-Curado et al [27]	2013	Distal ureteral	5 to 10 mm	A Dornier Compact Delta lithotripter	electromagnetic	60	Low	78	1.14	12 months	Moderate	Low
						80	Intermediate	72	1.56			
Salem et al [30]	2014	Renal	10 to 20 mm	Dornier Lithotripter S	electromagnetic	80	Intermediate	30	1.53	After max. 3 sessions	High	Moderate
						120	High	30	2.1			
Nguyen et al [28]	2015	Ureteral	N/A	Modified HM3 lithotripter	electrohydraulic	60	Low	127	N/A	3 months	Moderate	Low
						90	Intermediate	113	N/A			

a. Frequency ranges were defined as high-frequency (100–120 SWs/minute), intermediate-frequency (80–90 SWs/minute), and low-frequency (60–70 SWs/ minute).

b. Quality assessment was based on Cochrane’s risk of bias as a quality assessment tool for RCTs. If four or more domains are deemed “unclear” or “no,” the study was classified as having a high risk of bias. If two or three domains were deemed “unclear” or “no,” the study was classified as having a moderate risk of bias

c. Modified bias risk which excluded most common two biases (performance and detection biases), demonstrated that most of studies had low or moderate bias risk.

**Table 2:** Success and complications of enrolled studies.

Study	Frequency (SW/min)	Frequency Category^a^	No. of Patients	No. of Success (%)	Criterion of Success	No. of Complication (%)	Clavien grade			
							**I**	**II**	**III**	**IV**
Pace et al [19]	60	Low	111	82 (73.9)	< 5 mm	12 (10.8)	10	1	1	0
	120	High	109	66 (60.6)		21 (19.3)	19	1	1	0
Yilmaz et al [20]	60	Low	57	51 (89.5)	< 3 mm	0 (0)	0	0	0	0
	90	Intermediate	57	50 (87.7)		0 (0)	0	0	0	0
	120	High	56	41 (73.2)		1 (1.8)	1	0	0	0
Madbouly et al [5]	60	Low	76	75 (98.7)	< 2 mm	NA	N/A			
	120	High	80	72 (90.0)		NA	N/A			
Davenport et al [21]	60	Low	49	29 (59.2)	< 4 mm	5 (10.2)	N/A			
	120	High	51	31 (60.8)		4 (7.8)	N/A			
Li et al [29]	90	Intermediate	57	38 (66.7)	< 3 mm	5 (8.8)	5	0	0	0
	120	High	59	27 (45.8)		6 (10.2)	6	0	0	0
Honey et al [22]	60	Low	77	50 (64.9)	Stone free	9 (11.7)	5	3	1	0
	120	High	86	42 (48.8)		6 (7.0)	4	1	1	0
Koo et al [23]	70	Low	51	35 (68.6)	Stone free	NA	N/A			
	100	High	51	14 (27.5)		NA	N/A			
Mazzucchi et al [26]	60	Low	143	76 (53.1)	≤ 3 mm	7 (4.9)	6	1	0	0
	90	Intermediate	157	86 (54.8)		10 (6.4)	7	3	0	0
Chang et al [25]	60	Low	81	57 (70.4)	< 4 mm	NA	N/A			
	120	High	80	46 (57.5)		NA	N/A			
Ng et al [24]	60	Low	103	52 (50.5)	< 4 mm	14 (13.6)	14	0	0	0
	120	High	103	37 (35.9)		23 (22.3)	23	0	0	0
Anglada-Curado et al [27]	60	Low	78	78 (100)	Stone free	NA	N/A			
	80	Intermediate	72	67 (93.1)		NA	N/A			
Salem et al [30]	80	Intermediate	30	27 (90.0)	< 3 mm	4 (13.3)	0	4	0	0
	120	High	30	22 (73.3)		2 (6.7)	0	2	0	0
Nguyen et al [28]	60	Low	127	101 (79.5)	Stone free	13 (10.2)	6	2	1	4
	90	Intermediate	113	103 (91.2)		17 (15.0)	4	1	5	7

a. Frequency ranges were defined as high-frequency (100–120 SWs/minute), intermediate-frequency (80–90 SWs/minute), and low-frequency (60–70 SWs/ minute).

### Quality Assessment

**Fig 3:**
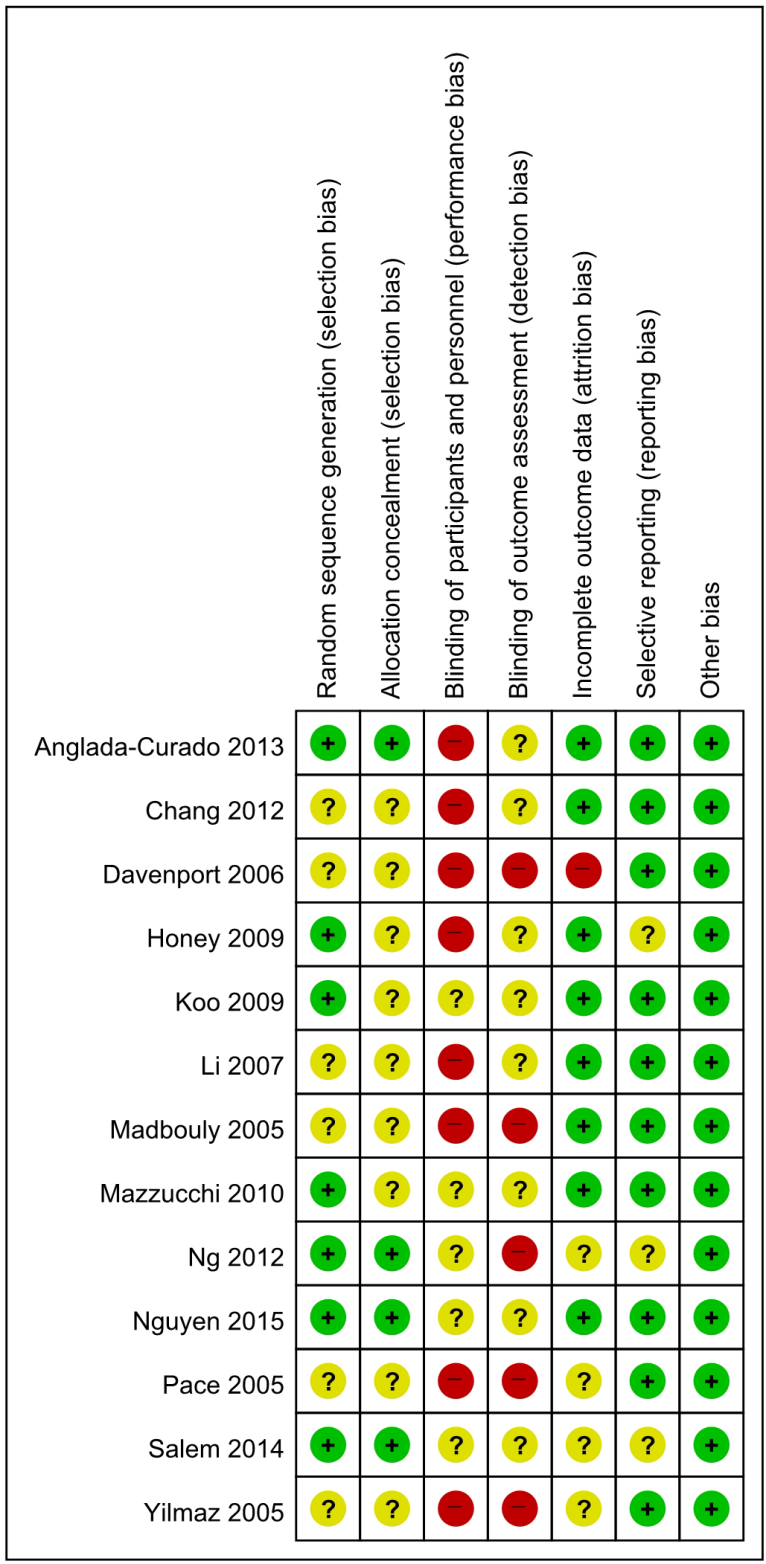
Risk-of-bias summary. Review author judgments for risk-of-bias items for each included study. Green, low risk of bias; Red, high risk of bias; Yellow, unclear of risk of bias.

Fig 3 presents details of quality assessment, as measured by the Cochrane Collaboration risk-of-bias tool. Seven trials exhibited a moderate risk of bias for all quality criteria and two studies were classified as having a high risk of bias (Table 1). The most common risk factor for quality assessment was the blinding of participants and personnel (performance bias), and the second most common parameter concerned the blinding of outcome assessment (detection bias). These biases are related to study design, which can be performed in single-blinded or non-blinded formats.

### Heterogeneity and Inconsistency Assessment, and Publication Bias

**Fig 4:**
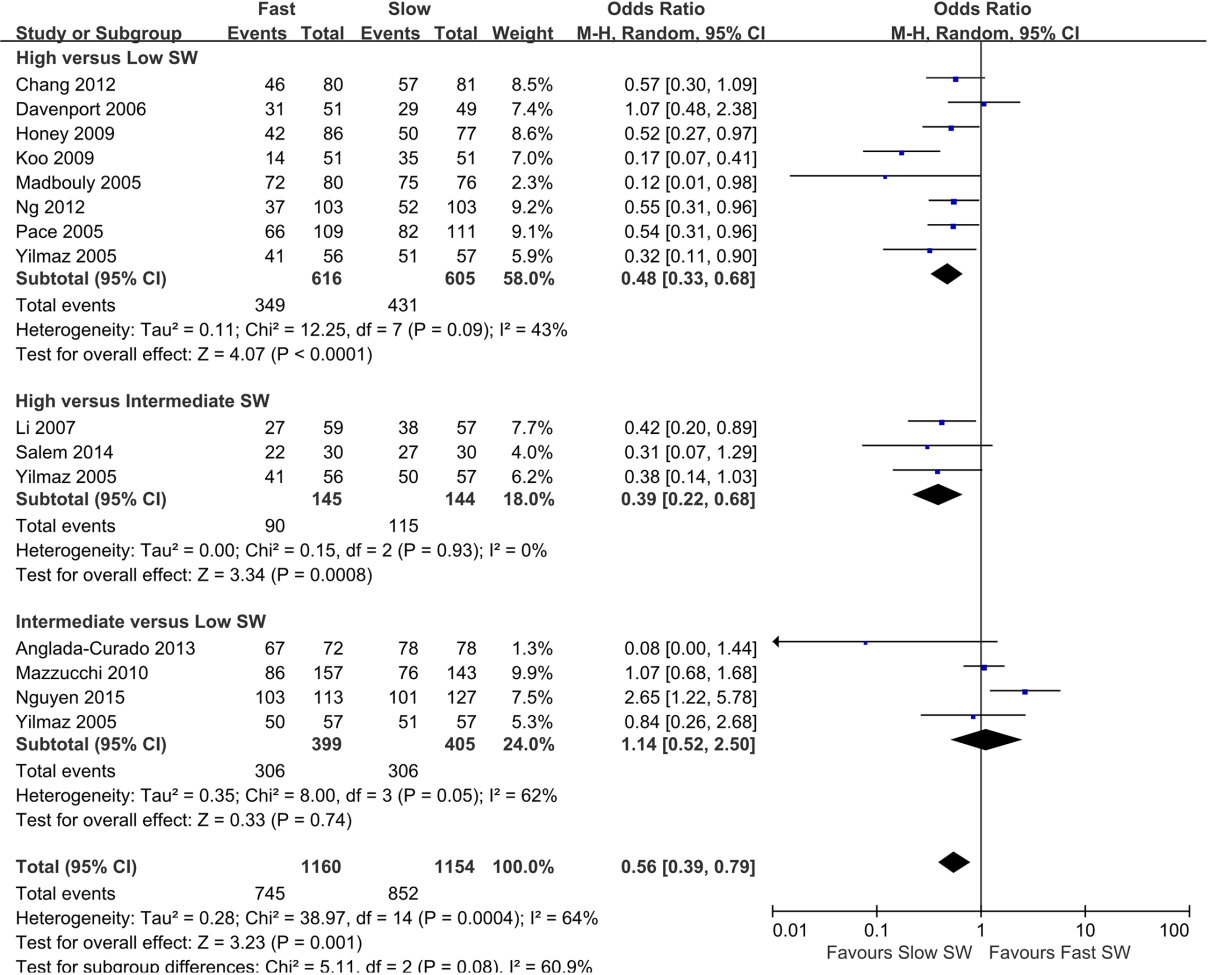
Pairwise meta-analysis of success rate in each SW-frequency range following SWL. Pooled data that assessed overall success showed a significantly lower rate of overall success in the high-frequency SWL versus low-frequency SWL groups (OR 0.48; 95% CI 0.33–0.68; P<0.001), and in the high-frequency SWL versus intermediate-frequency SWL groups (OR 0.39; 95% CI 0.22–0.68; P<0.001). However, forest plots showed no significant difference in success rate between intermediate-frequency SWL versus low-frequency SWL.

**Fig 5:**
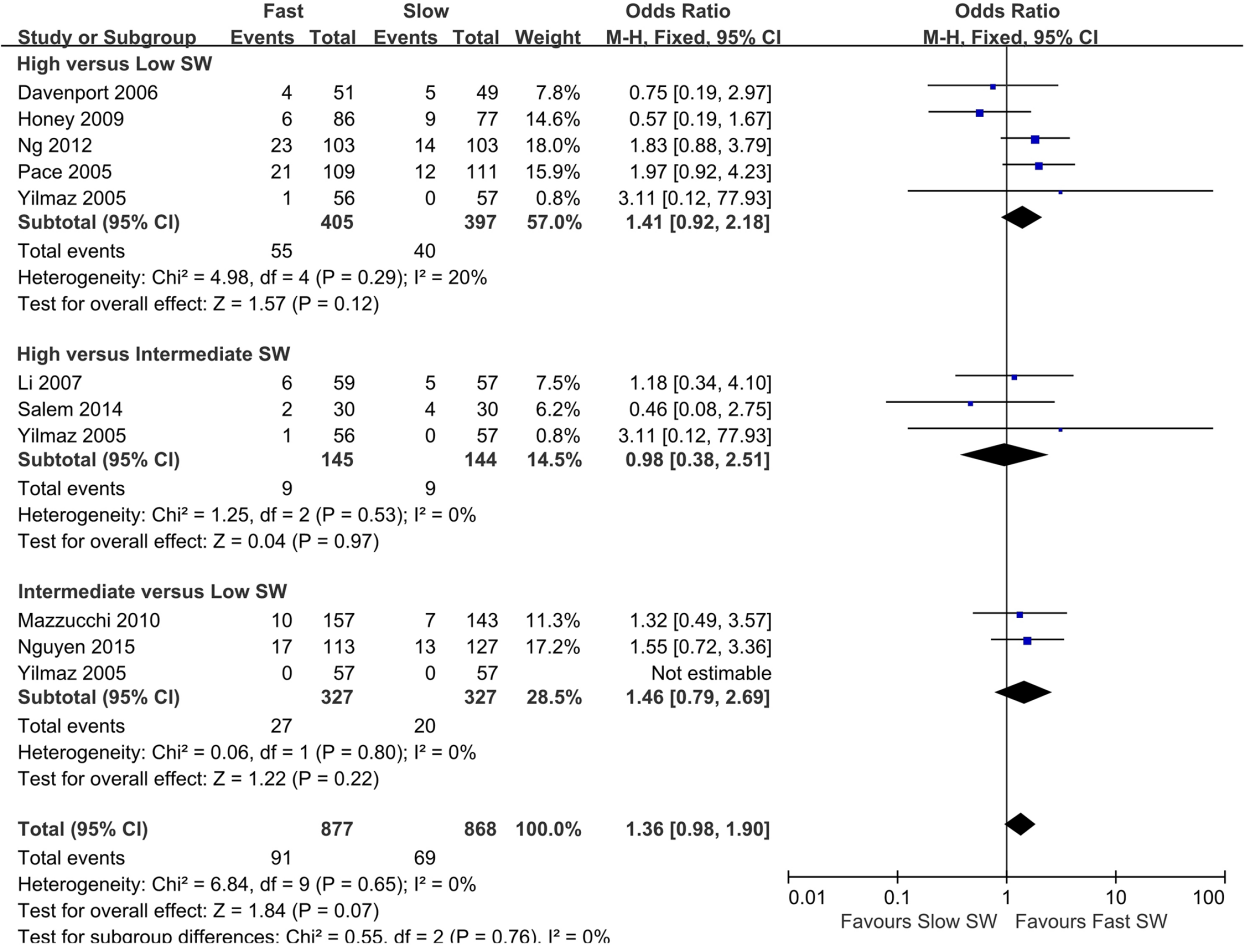
Pairwise meta-analysis for complication rate in each shock wave frequency range following SWL. Forest plots for complication rate demonstrated no significant difference between high-frequency SWL versus low-frequency SWL (OR 1.41; 95% CI 0.92–2.18; P = 0.12), high-frequency SWL versus intermediate-frequency SWL (OR 0.98; 95% CI 0.38–2.51; P = 0.97), or intermediate-frequency SWL versus low-frequency SW (OR 1.46; 95% CI 0.79–2.69; P = 0.22).

**Fig 6:**
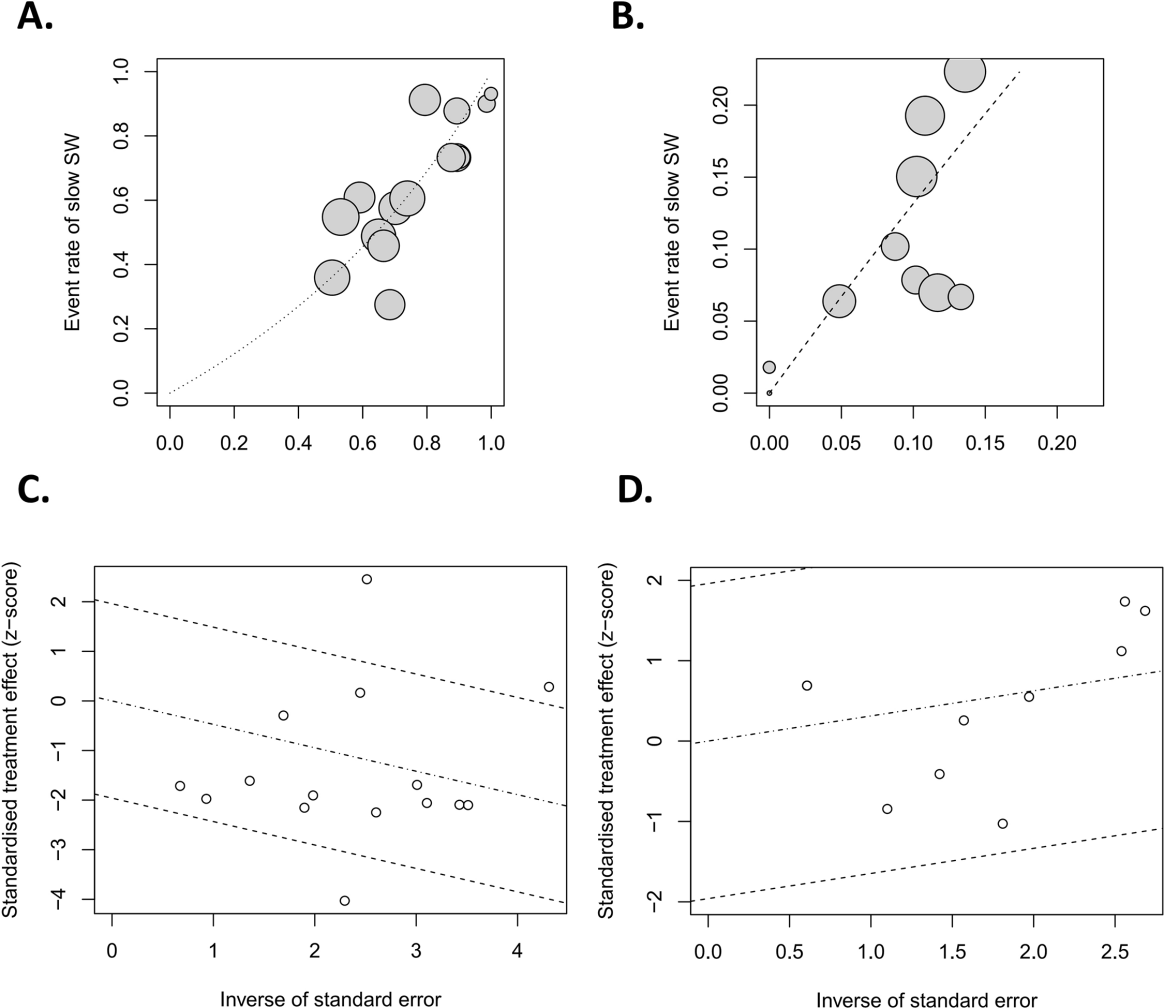
**L’Abbe plots of success (A) and complication rates (B), and Galbraith’s radial plots of success (C) and complication rates (D).** In the L’Abbe plots, the success rate showed comparable inter-study heterogeneity, and the complication rate had slight heterogeneity. Radial plots revealed that three studies were located outside of the 95% CI of linear prediction; however, the complication rates of all studies were inside of the 95% CI of linear prediction.

Forest plots of the pairwise meta-analyses of SWL success and complications are shown in Figs 4 and 5, respectively. A heterogeneity test for SWL success rate showed the following: χ^2^ = 46.78 with 14 df (P<0.001), and I^2^ = 70.0% in the total test for success rate; and χ^2^ = 8.35 with 2 df (P = 0.02) and I^2^ = 76.1% in the test for subgroup differences. Thus, in success-rate analyses, the random-effect models were applied using the Mantel–Haenszel method. In the analysis of SWL complication rate, a heterogeneity test also demonstrated homogeneity with χ^2^ = 6.58 with 8 df (P = 0.58) and I^2^ = 0% in total test, and χ^2^ = 0.53 with 2 df (P = 0.77) and I^2^ = 0% in the test for subgroup differences. Because there was no heterogeneity in forest plots for complication rate, the fixed-effect models were applied using the Mantel–Haenszel method. In the L’Abbe plot, the success rate showed comparable inter-study heterogeneity (Fig 6A), and the complication rate had slight heterogeneity in the L’Abbe plot (Fig 6B). Radial plots revealed that three studies were located outside of the 95% CI of linear prediction (Fig 6C); however, the complication rates of all studies were inside of the 95% CI of linear prediction (Fig 6D).

**Fig 7:**
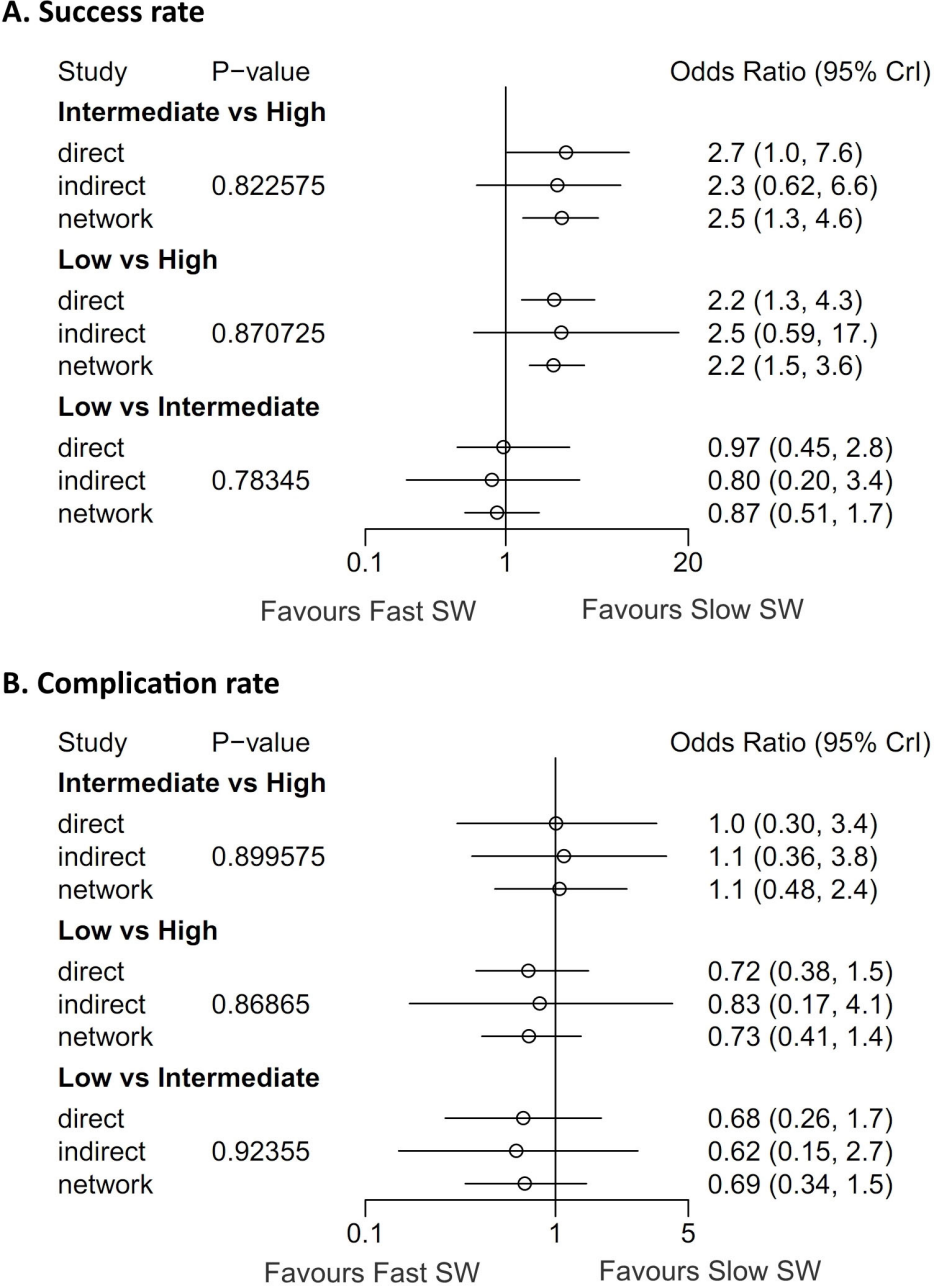
Network meta-analysis for success and complication rates according to SWL frequency and node-splitting analyses of inconsistency. (A) The success rates of low- (OR 2.2; 95% CI 1.5–3.6) and intermediate-frequency SWL (OR 2.5; 95% CI 1.3–4.6) were higher than high-frequency SWL on the network analyses. (B) In terms of the complication rate, there were no differences across all SWL frequency groups. On node-splitting analysis, no comparisons demonstrated inconsistency between direct and indirect comparison in terms of success and complication rates. P-value: inconsistency p-values for each split comparison. Direct: direct comparison between two treatments. Indirect: indirect comparison between two treatments. Network: network meta-analysis between two treatments

**Fig 8:**
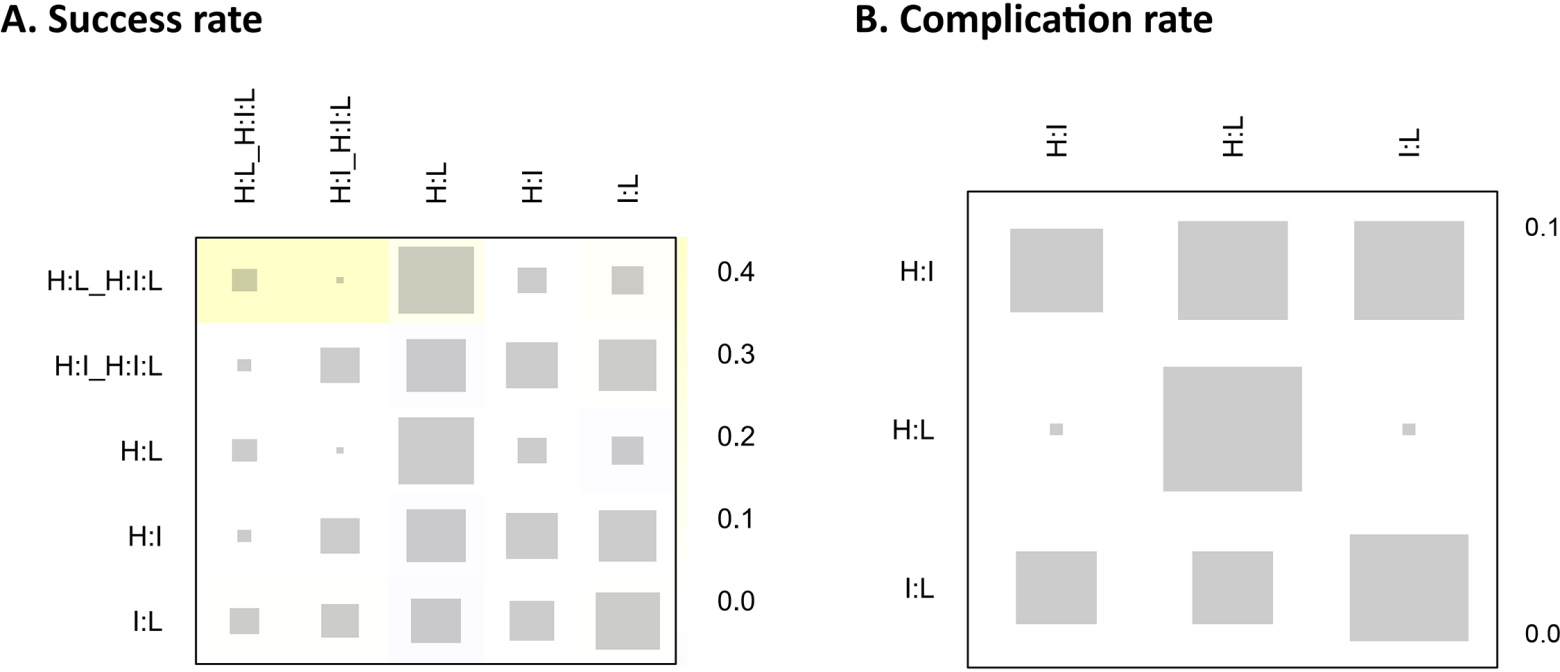
Net-heat plot for inconsistency. The net-heat plot also showed that there was only slight inconsistency throughout the entire network in terms of the (A) success rate; however, there was no inconsistency in terms of the (B) complication rate.

On the assessments of homogeneity and consistency, the Q statistic showed inconsistency throughout the entire network (P = 0.073) and within designs (P = 0.0263); however, no inconsistency was found between designs (P = 0.8434) on the success rate analysis. The Q statistic for the entire network between designs (after detaching single designs) demonstrated no inconsistencies among all comparisons and triple comparisons with loops on success rate analysis. Additionally, on complication analysis, there was no inconsistency in the Q statistic (Table 3). On node-splitting analysis, no comparison demonstrated inconsistency among direct, indirect, and network comparisons (Fig 7). The net-heat plot also showed that there was only slight inconsistency throughout the entire network in terms of the success rate (Fig 8A), and there was no inconsistency in terms of the complication rate (Fig 8B).

**Table 3:** Design-based decomposition of Cochran’s Q in network meta-analyses of success and complication rates.

	Success rate			Complication rate		
	Q	df	P-value	Q	df	P-value
	Q statistic to assess homogeneity / consistency					
**Whole network**	19.7	12	0.073	5.46	6	0.486
**Within designs**	18.87	9	0.026	5.46	5	0.362
**Between designs**	0.83	3	0.843	0	1	0.969
	Between-designs Q statistic after detaching single designs					
**High vs. Intermediate**	0.15	1	0.697	0.7	1	0.401
**High vs. Low**	11.5	6	0.074	4.76	4	0.313
**Intermediate vs. Low**	7.23	2	0.027	0	0	NA
	Between-designs Q statistic after detaching single designs					
**High vs. Intermediate**	0.82	2	0.662	0	0	NA
**High vs. Low**	0.68	2	0.712	0	0	NA
**Intermediate vs. Low**	0.77	2	0.680	0	0	NA
**High vs. Intermediate vs. Low**	0	1	0.975	NA	NA	NA
	Q statistic to assess consistency under the assumption of a full design-by-treatment interaction random effects model					
**Between designs**	0.48	3	0.924	0	1	0.982

**Fig 9:**
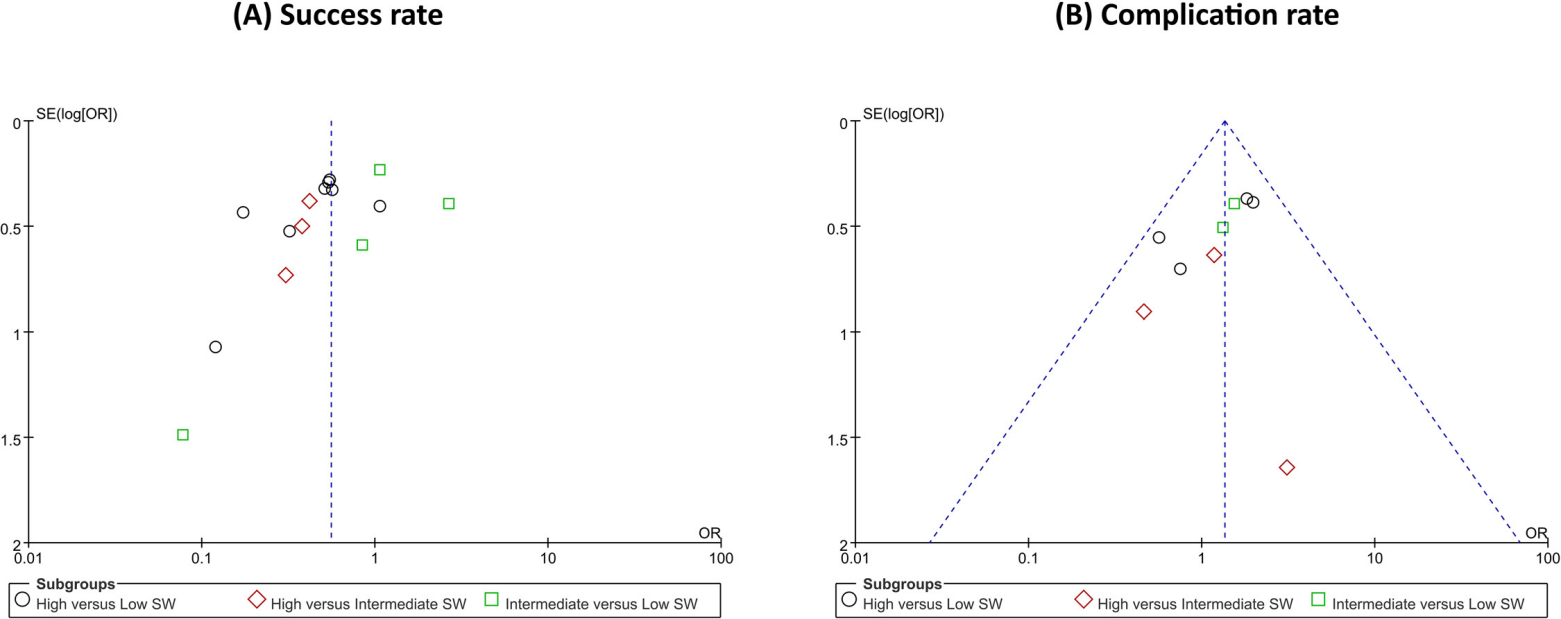
**Funnel plots of success (A) and complication rates (B).** Little evidence of publication bias was demonstrated by visual or statistical examination of the funnel plots.

Funnel plots from pairwise meta-analyses are demonstrated in Fig 9; however, it was difficult to assess publication bias using a limited number of studies, although some degree of bias is suspected. In addition, the Begg and Mazumdar rank correlation tests and Egger’s regression intercept tests for the success rate showed publication bias (P = 0.046 and P = 0.005, respectively). However, for the complication rate, there was no publication bias based on the two tests (P = 0.273 and P = 0.485, respectively).

### Pairwise Meta-analysis of Success and Complication Rates of SWL

Pooled data that assessed overall success showed a significantly lower rate of overall success with high-frequency SWL versus low-frequency SWL (OR 0.48; 95% CI 0.33–0.68; P<0.001), and high- versus intermediate-frequency SWL (OR 0.39; 95% CI 0.22–0.68; P<0.001). However, forest plots showed no significant difference between intermediate- versus low-frequency SWL for success rate (OR 1.14; 95% CI 0.52–2.50; P = 0.74). The total test demonstrated that low-frequency SWL produced more favorable outcomes than high-frequency SWL (OR 0.56; 95% CI 0.39–0.79; P = 0.001) (Fig 4). Forest plots for complication rate demonstrated no significant difference between high- versus low-frequency SWL (OR 1.41; 95% CI 0.92–2.18; P = 0.12), high- versus intermediate-frequency SWL (OR 0.98; 95% CI 0.38–2.51; P = 0.97), or intermediate- versus low-frequency SWL (OR 1.46; 95% CI 0.79–2.69; P = 0.22). The final pooled data for complication rate showed no significant difference between low-frequency SWL versus high-frequency SWL (OR 1.36; 95% CI 0.98–1.90; P = 0.07) (Fig 5).

**Fig 10:**
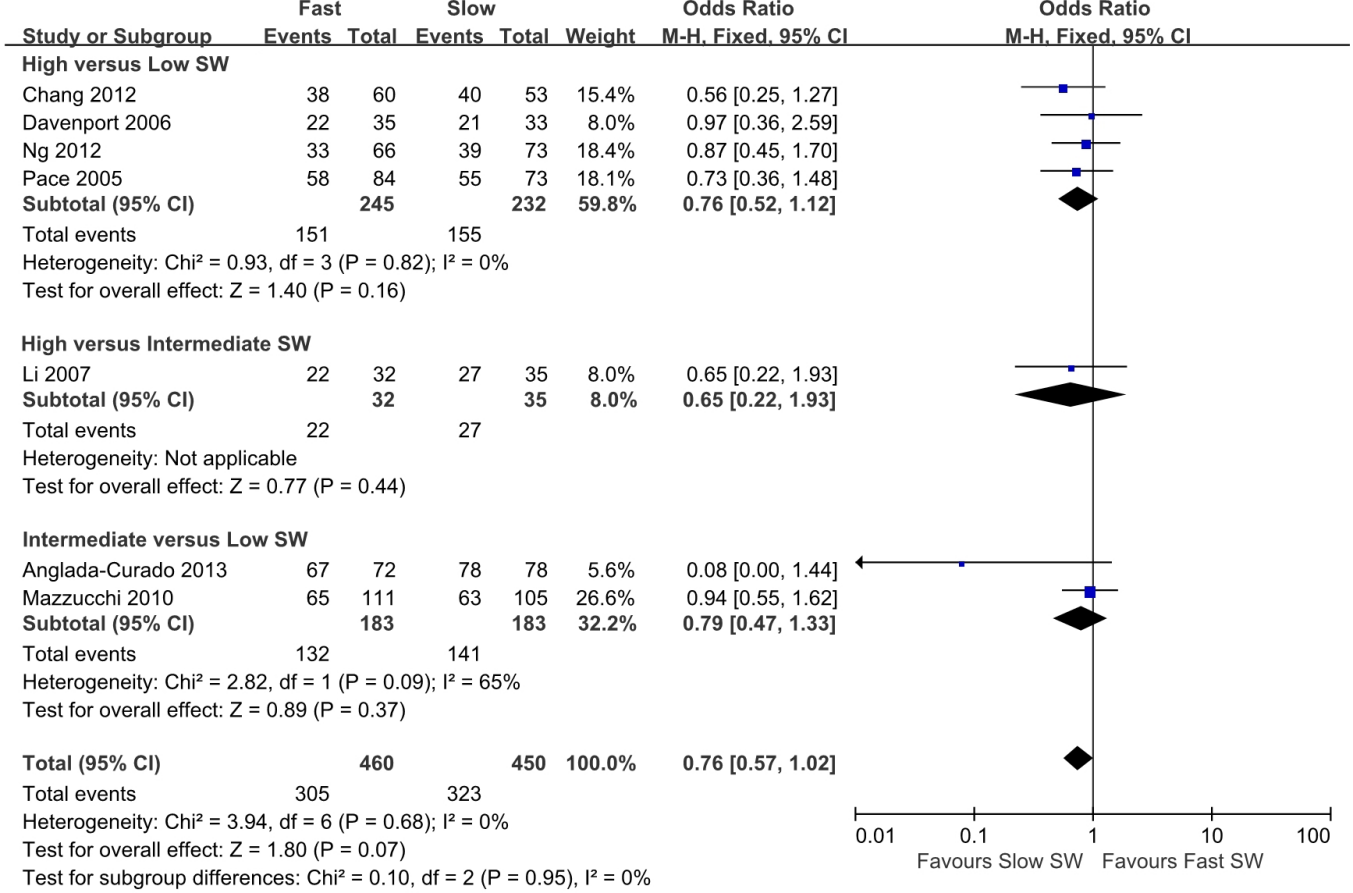
Pairwise meta-analysis of success rates in each SW-frequency range following SWL in stones <10 mm. There were no differences in the success rate for stones less than 10 mm.

**Fig 11:**
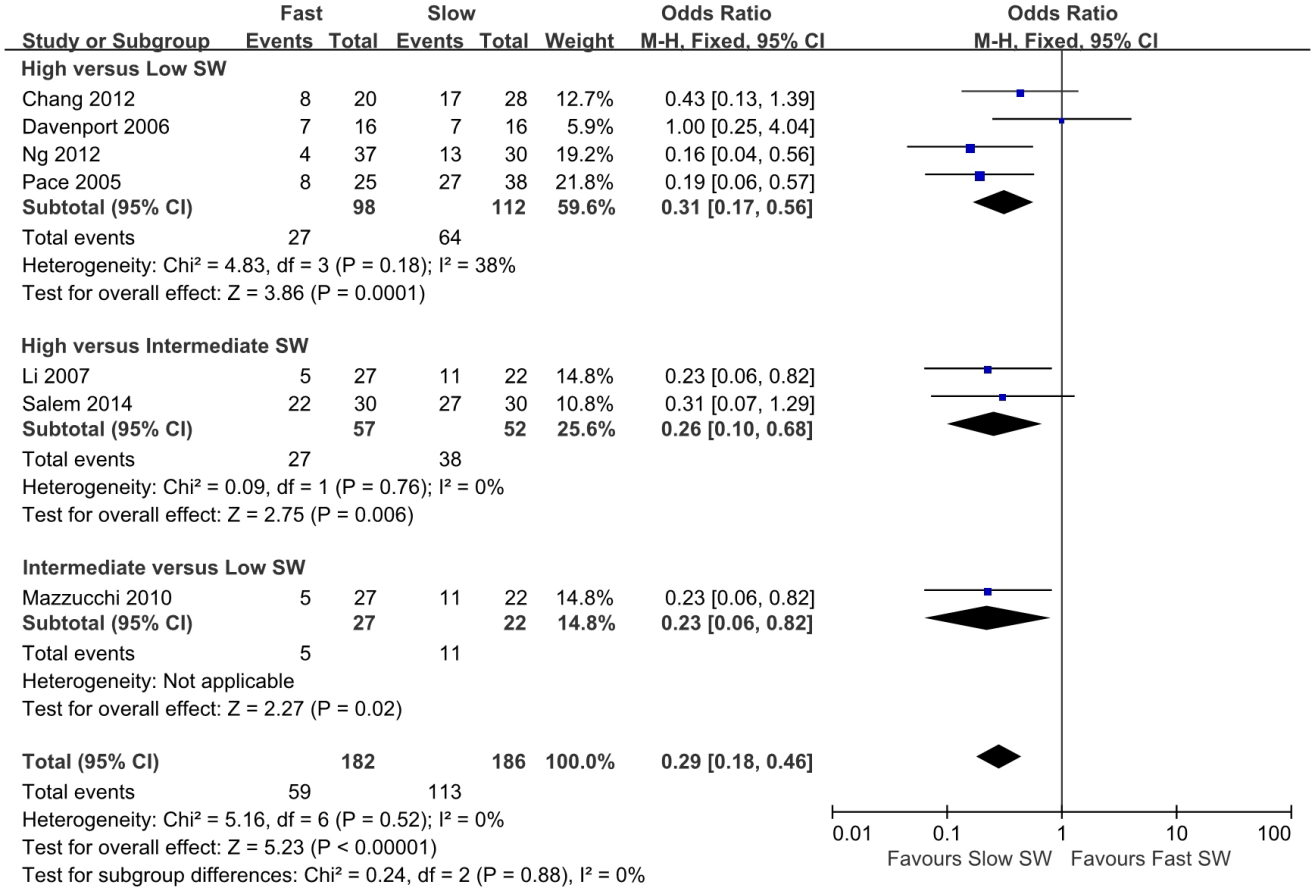
Pairwise meta-analysis of success rates in each SW-frequency range following SWL in stones ≥10 mm. The success rate of low SW was higher than that of high SW (OR 0.31; 95% CI 0.17–0.56; P<0.001). Intermediate SW analyses that included only one study demonstrated success rates that were higher than high SW and lower than low SW.

**Fig 12:**
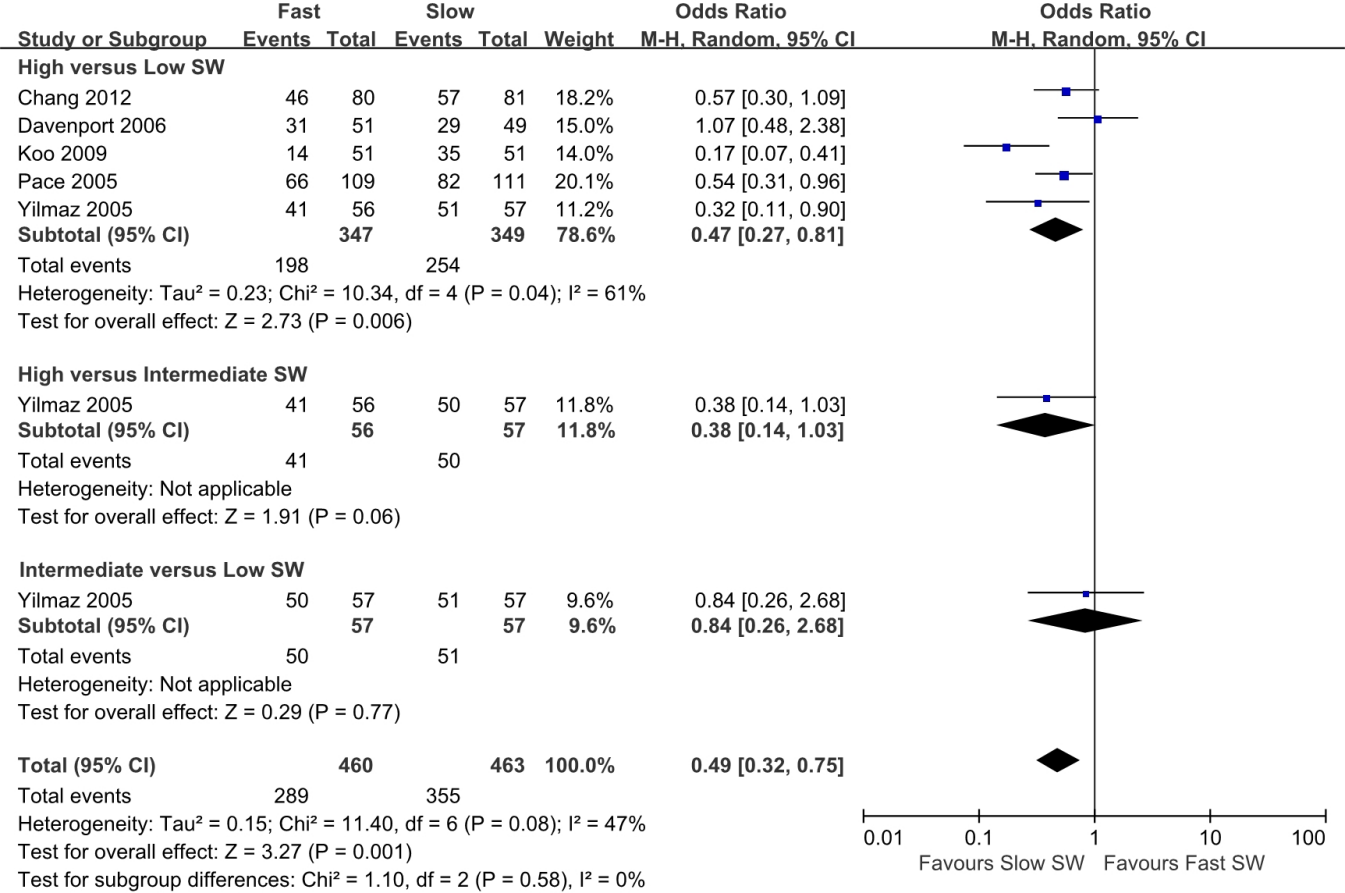
Pairwise meta-analysis of success rates in each SW-frequency range following SWL for renal stones. The success rate of low SW was higher than that of high SW (OR 0.47; 95% CI 0.27–0.81; P = 0.006)

On subgroup analyses, there were no differences in the success rate for stones of less than 10 mm (Fig 10). However, for stones of 10 mm or greater, the success rate of low SW was higher than high SW (OR 0.31; 95% CI 0.17–0.56; P<0.001). Intermediate SW analyses that included only one study demonstrated success rates that were higher than high SW and lower than low SW (Fig 11). In renal stones, the success rate of low SW was higher than that of high SW (OR 0.47; 95% CI 0.27–0.81; P = 0.006; Fig 12).

### Network Meta-analysis of Success and Complication Rates of SWL

**Fig 13:**
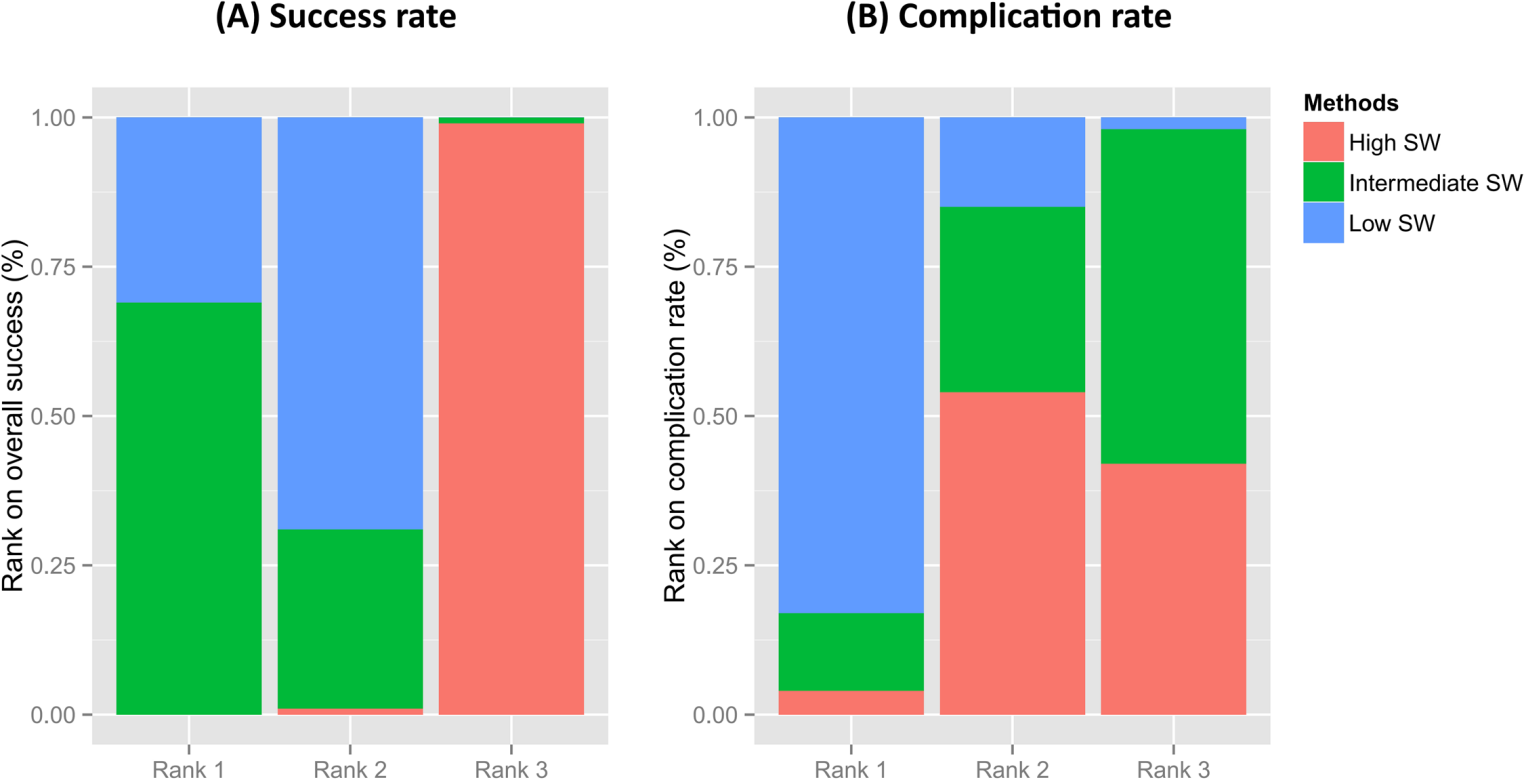
Rank-probability test of network meta-analyses. Intermediate-frequency SWL had the highest rank for success rate, followed by low- and high frequency SWL (**A**) Low-frequency SWL also ranked highest for low complication rate, (B) High- and intermediate-frequency SWL were ranked lowest for low complication rate.

On network meta-analyses, the success rates of low- (OR 2.2; 95% CI 1.5–2.6) and intermediate-frequency SWL (OR 2.5; 95% CI 1.3–4.6) were higher than high-frequency SWL (Fig 7A). Forest plots from the network meta-analysis also showed no significant differences in the success rate between low-frequency SWL versus intermediate-frequency SWL (OR 0.87; 95% CI 0.51–1.7). In complication rate, there were no differences across all SW frequency categories (Fig 7B). In the rank-probability test, intermediate-frequency SWL had the highest rank for success rate, followed by low- and high-frequency SWL (Fig 13). Low-frequency SWL was also ranked highest for low complication rate. High- and intermediate-frequency SWL were ranked lowest for low complication rate. A P-score test using a frequentist method to rank treatments in the network demonstrating intermediate-frequency SWL (P-score; 0.928) was superior to low- (P-score 0.572) and high-frequency SWL (P-score 0) in terms of the success rate [31]. Regarding the complication rate, the P-scores of low-, intermediate- and high-frequency SWL were 0.933, 0.217, and 0.350, respectively.

### Discussion

With SWL implementation for curing urinary stone disease, balancing increased success rate with decreased complication remains a perennial problem for urologists. Many studies have shown that urinary stone size and location, lithotripter type, and operator skill are factors that influence the success rate of SWL treatment. Moreover, to maximize efficacy and SWL success rate, studies have focused on the effects of modifying controllable factors, such as SW frequency, maximum voltage escalation rate, and total number of shocks. In general, altering these factors is likely to illuminate an inverse relationship between the SWL efficacy and occurrence of complications, depending on the magnitude of change. Thus, it is important to determine the optimal range of these parameters that satisfies both efficacy and stability. Especially, many researchers have been interested in efficacy and stability resulting from using different SW frequencies.

Since the introduction of SWL, the most commonly used SW frequency has been 120 SW/minute. However, *in vivo* and *in vitro* experiments and clinical research from the mid-1990s showed that reducing the SW frequency increases stone fragmentation [2–4]. Since then, several RCTs have begun investigating the effect of decreasing SW frequency on procedure efficacy. The first RCTs were studies conducted by Madbouly et al [5] and Pace et al [19]. These groups compared the treatment outcome of SWL at 120 SW/minute versus 60 SW/minute, and both studies showed better outcomes using low-frequency SWL. Since then, several studies have further compared the influence of delivering high-frequency SWs versus low-frequency SWs, and confirmed improved results with low-frequency SWL [20,22–25].

Though the mechanism underlying the relative benefit of low-frequency SWL remains uncertain, several hypotheses have been generated regarding cavitation bubble generation. The first possible mechanism involves decreased mismatch of acoustic impedance [32]. This hypothesis states that higher SW delivery rates result in less effective SW energy transmission due to acoustic scattering and dampening, likely because of increased cavitation bubble production [33]. The shorter interval between SW pulses at higher delivery rates, the more bubbles are generated. Although cavitation bubbles on stone surfaces contribute to stone fragmentation, continuous cavitation bubbles act as a barrier to SW energy transmission by forming bubble clouds, thereby reducing stone fragmentation effects. Thus, slower SW delivery rate removes the bubble barrier extent on the stone surface and supports better cluster dynamics that facilitate superior fragmentation [34]. Based on these hypothesized mechanisms, most studies that compared low-frequency versus high-frequency SWL have shown better results with low-frequency SW delivery. However, in the study conducted by Davenport et al [21], there was no difference in effectiveness between these two broad SWL frequency groups. The authors believed that the main reason for the similar results was that their studies were performed only on patients with small stones that were limited to the kidney. Our comprehensive meta-analyses of all reported RCTs to date confirm that performing SWL with a lower SW rate results in better outcomes than with a higher SW rate.

Though low-frequency SWL is more effective than high-frequency SWL, the main drawback is that it takes a longer time. Furthermore, although SWL approaches differ among countries and global regions, cost effectiveness aspects of lower SW delivery rates should be considered. Thus, several researchers have begun to take interest in using intermediate-frequency SWL. Li et al [29] reported that in 116 patients with renal or ureteral stones, 90 SW/minute led to better treatment outcomes than using 120 SW/minute, and the success rate was particularly increased in patients having a stone >10 mm. Salem et al [30] performed a study conducted on pediatric patients having larger renal stones (size range 10‒20 mm), and intermediate-frequency SWL had significantly better treatment outcomes than using high-frequency SWL. In our meta-analysis, the intermediate SW rate had significantly better efficacy than high-frequency SWL.

Though differences in efficacy between high-frequency versus intermediate- and low-frequency SWL are obvious, there remains controversy about the comparative efficacy of intermediate- versus low-frequency SWL. Yilmaz et al [20] observed no difference in treatment outcome when comparing 90/minute and 60/minute SW rates, but the 90 SW rate was considered to be the optimal frequency because of reduced procedural duration. Mazzucchi et al [26] reported no difference in the stone-free rate between two groups: one using 3000 total pulses at the 60 SW/minute rate, and the other implementing 4000 pulses at the 90 SW/minute rate. In a study of 154 patents with distal ureter stones, the 60 SW/minute rate showed better outcomes than the 80 SW/minute rate [27]. However, another study observed that the 90 SW/minute rate showed better outcomes than the 60 SW/minute rate [28]. Those researchers presented a different view than previous theories, and assumed that an increased SW delivery rate enhances cavitation bubble production on the stone surface, which enhanced fragmentation. In our meta-analysis, there was no significant efficacy difference between the intermediate-frequency and low-frequency groups. However, in the rank test, the intermediate SW rate was ranked highest for success. Because it remains difficult to conclusively determine the treatment outcome of the intermediate versus low SW rate with the existing data, large-sample RCTs should be performed.

We also performed a subgroup analysis for more stratified outcome data by size and location. There were several RCTs divided by 10-mm stone size, and there were no differences in the success rate for stones less than 10 mm. However, for stones of 10 mm or greater, the success rate of low SW was higher than high SW (five studies). The effect of SWL can be enhanced with larger stones, where SW energy is more effectively delivered to the stone surface, and this may be impacted by SW frequency. However, as only three studies included intermediate SW for stones of 10 mm or greater, the clinical significance might be very weak for intermediate- versus high-frequency groups and intermediate- versus low-frequency groups.

As an indicator of treatment success, the complication rate is as important as the efficacy rate. Though decreased SW frequency may reduce incidental damage because of the decreased total number of shocks, it concurrently shows more effectiveness in stone fragmentation due to the altered cavitation bubble dynamics. Capillary rupture can be avoided by allowing more time for bubbles to dissipate between shocks [35]. Nonetheless, our pairwise and network meta-analyses indicated that there is no significant difference in the complication rate among the three SW-frequency groups. The reason might be because most studies have shown very low overall complication rates of SWL, without reference to using any specific SW frequency, which reflects the overall safety of the SWL approach. Therefore, although there might be no need to place great importance on potential complications when determining the optimal frequency for SWL, our rank-test results showed that low SW frequency was ranked highest for the low complication rate. Thus, an additional study on complications of SWL depending on applied SW frequency is needed.

One limitation of our meta-analysis is that we did not assess the impact of the total number of SWs delivered as a function of SW frequency, which may have introduced critical bias. In the results, there was another limitation in that the success rate showed a degree of heterogeneity in the forest, L’Abbe, and radial plots. Thus, we used a random effect model to analyze the outcome in terms of the success rate. Additionally, our study was also susceptible to a degree of publication bias. However, Sutton et al. reviewed 48 articles from the Cochrane Database of Systematic Reviews and showed that publication bias and related biases were common within their meta-analysis sample [36]. They found that these biases did not affect the conclusions in most cases. Similar to heterogeneity, inconsistency is caused by effect modifiers and specifically by an imbalance in the distribution of effect modifiers in the direct and indirect evidence from the network meta-analysis [37,38]. In our study, there was only slight inconsistency on network analyses using Cochran’s Q statistic, node-splitting analysis, and net-heat plots. Thus, in our results, there was agreement between direct and indirect comparison. Despite these limitations, our study has sufficient value as a meta-analysis because it spans studies performed over a longer period than previous analyses [39]. Moreover, the current study is unique as it applied network meta-analysis methods in order to enhance the statistical confidence and overcome the limitations of pairwise meta-analysis.

## Conclusions

Network meta-analysis of published RCT data on SWL frequency confirms that intermediate-frequency and low-frequency SWL show better treatment outcomes than high-frequency SWL in terms of both efficacy and complication rates. However, we require more data to conclusively determine whether intermediate versus low SW rates produce optimal results in SWL.

## Supporting Information

S1 Table Preferred Reporting Items for Systematic Reviews and Meta-Analyses (PRISMA) statement. (DOC)

S2 Table Search strategy in PubMed. (DOC)
