# Supplementary material for: Comparison of High, Intermediate, and Low Frequency Shock Wave Lithotripsy for Urinary Tract Stone Disease: Systematic Review and Network Meta-Analysis
Source: PLoS One. 2016 Jul 7;11(7):e0158661. doi: 10.1371/journal.pone.0158661 (PMC4936716; doi:10.1371/journal.pone.0158661)
Supplement: S2 Table — (DOC) [file pone.0158661.s002.doc]

| S2 Table. Search strategy in PubMed | | |
| --- | --- | --- |
| Search | Query | Items found |
| #1 | Search **(extracorporeal shock wave lithotripsy) OR shock wave lithotripsy** | 11468 |
| #2 | Search **(frequency) OR rate** | 3743581 |
| #3 | Search **((renal stone) OR ureter stone) OR urolithiasis** | 37182 |
| #4 | Search **((success rate) OR stone-free) OR complication** | 258765 |
| #5 | Search **randomized controlled trial** | 501055 |
| #6 | Search **((((#1) AND #2) AND #3) AND #4) AND #5** | 149 |
